# Supplementary material for: Antimicrobial and Antibiofilm Properties of Latvian Honey against Causative Agents of Wound Infections
Source: Antibiotics (Basel). 2023 Apr 26;12(5):816. doi: 10.3390/antibiotics12050816 (PMC10215129; doi:10.3390/antibiotics12050816)
Supplement: Supplementary file 1 [file antibiotics-12-00816-s001.zip › Supplementary material Figure S1.pdf]

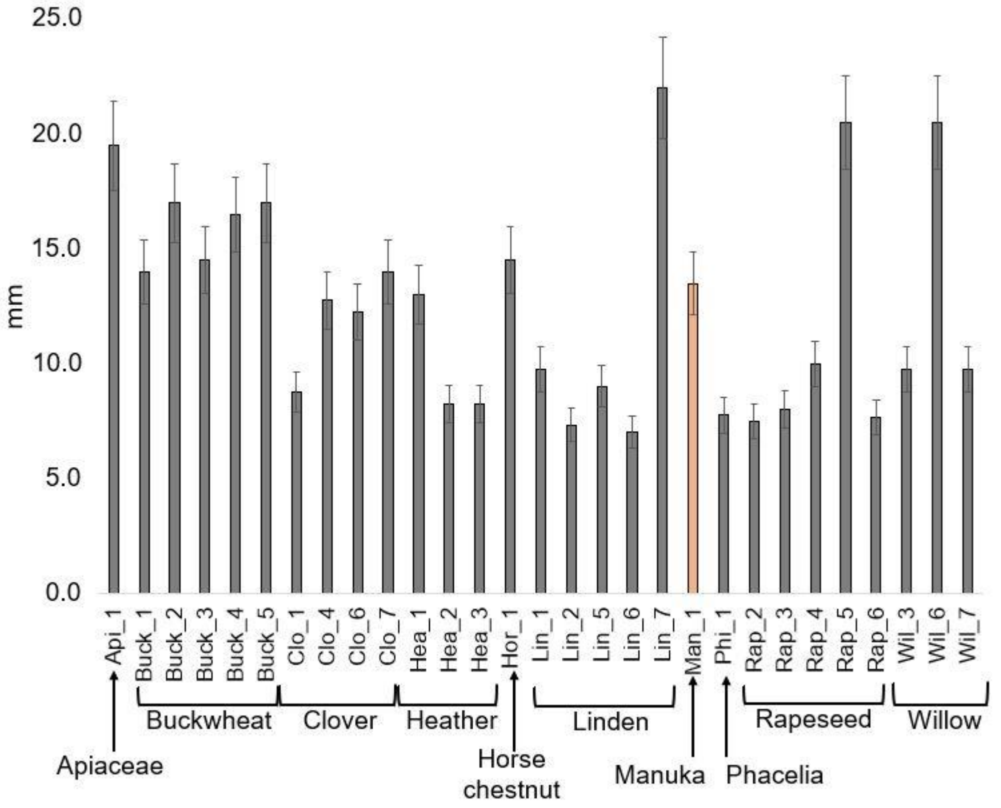

25.0
20.0
15.0
10.0
5.0
0.0
mm
Api_1
Buck_1
Buck_2
Buck_3
Buck_4
Buck_5
Clo_1
Clo_4
Clo_6
Clo_7
Hea_1
Hea_2
Hea_3
Hor_1
Lin_1
Lin_2
Lin_5
Lin_6
Lin_7
Man_1
Phi_1
Rap_2
Rap_3
Rap_4
Rap_5
Rap_6
Wil_3
Wil_6
Wil_7
Buckwheat
Clover
Heather
Linden
Rapeseed
Willow
Apiaceae
Horse chestnut
Manuka
Phacelia


**(a)**

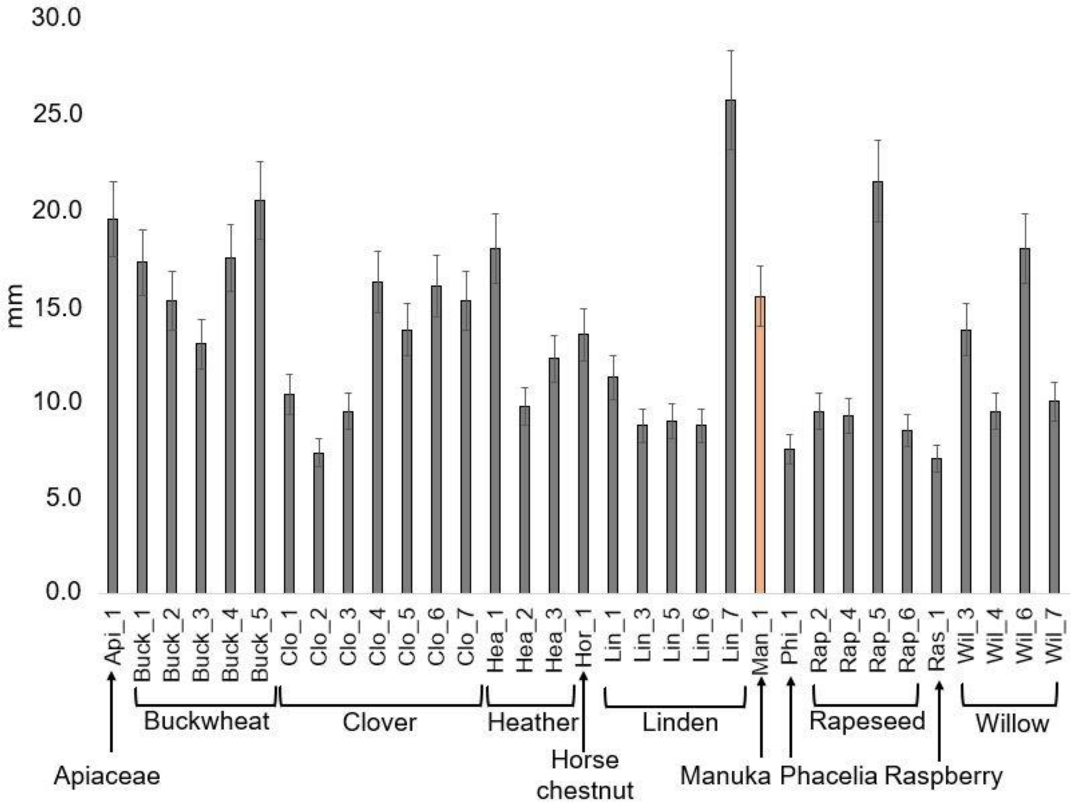

30.0
25.0
20.0
15.0
10.0
5.0
0.0
mm
Api_1
Buck_1
Buck_2
Buck_3
Buck_4
Buck_5
Clo_1
Clo_2
Clo_3
Clo_4
Clo_5
Clo_6
Clo_7
Hea_1
Hea_2
Hea_3
Hor_1
Lin_1
Lin_3
Lin_5
Lin_6
Lin_7
Man_1
Phi_1
Rap_2
Rap_4
Rap_5
Rap_6
Ras_1
Wil_3
Wil_4
Wil_6
Wil_7
Buckwheat
Clover
Heather
Linden
Rapeseed
Willow
Apiaceae
Horse chestnut
Manuka
Phacelia
Raspberry


**(b)**

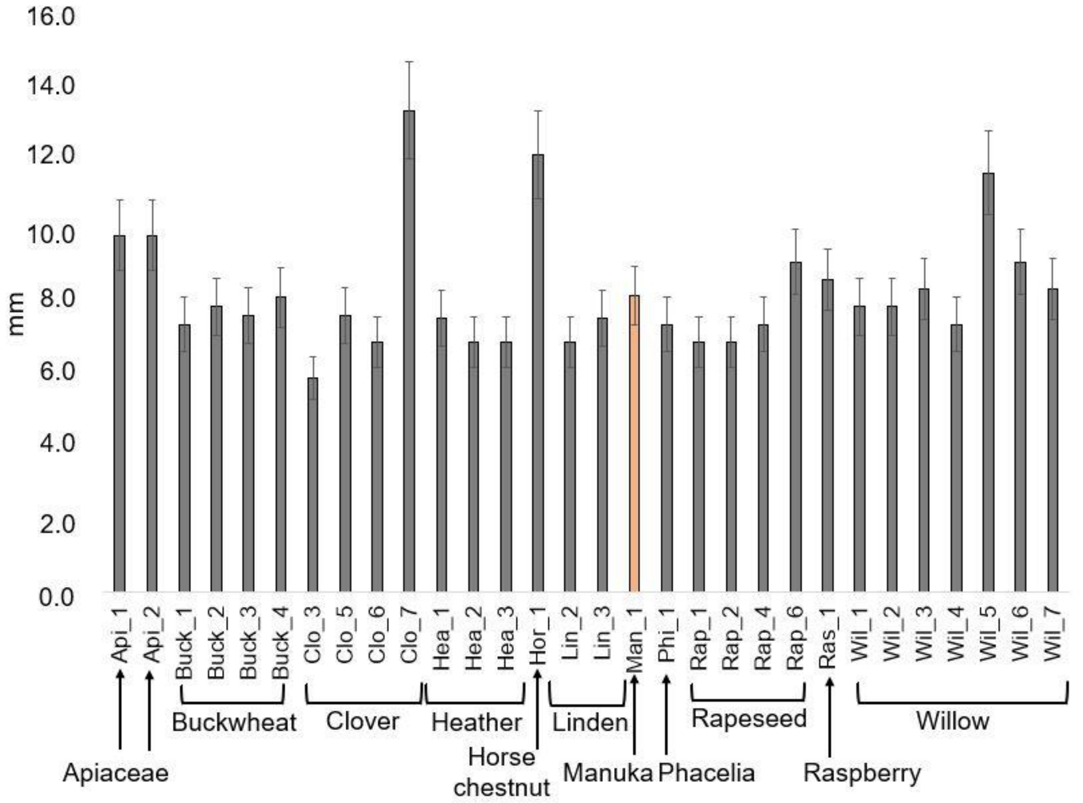

mm
0.0
2.0
4.0
6.0
8.0
10.0
12.0
14.0
16.0
Api_1
Api_2
Buck_1
Buck_2
Buck_3
Buck_4
Clo_3
Clo_5
Clo_6
Clo_7
Hea_1
Hea_2
Hea_3
Hor_1
Lin_2
Lin_3
Man_1
Phi_1
Rap_1
Rap_2
Rap_4
Rap_6
Ras_1
Wil_1
Wil_2
Wil_3
Wil_4
Wil_5
Wil_6
Wil_7
Apiaceae
Buckwheat
Clover
Heather
Horse chestnut
Linden
Manuka
Phacelia
Rapeseed
Raspberry
Willow


**(c)**

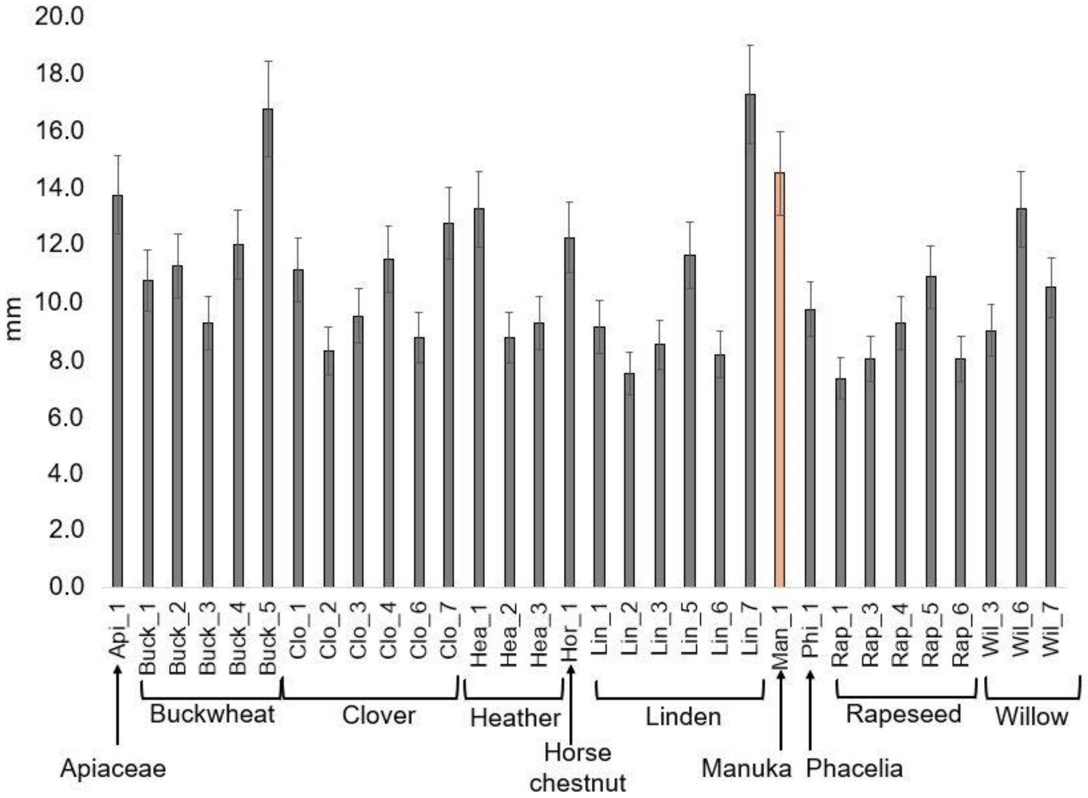

mm
0.0
2.0
4.0
6.0
8.0
10.0
12.0
14.0
16.0
18.0
20.0
Api_1
Buck_1
Buck_2
Buck_3
Buck_4
Buck_5
Clo_1
Clo_2
Clo_3
Clo_4
Clo_6
Clo_7
Hea_1
Hea_2
Hea_3
Hor_1
Lin_1
Lin_2
Lin_3
Lin_5
Lin_6
Lin_7
Man_1
Phi_1
Rap_1
Rap_3
Rap_4
Rap_5
Rap_6
Wil_3
Wil_6
Wil_7
Apiaceae
Buckwheat
Clover
Heather
Horse chestnut
Linden
Manuka
Phacelia
Rapeseed
Willow


**(d)**

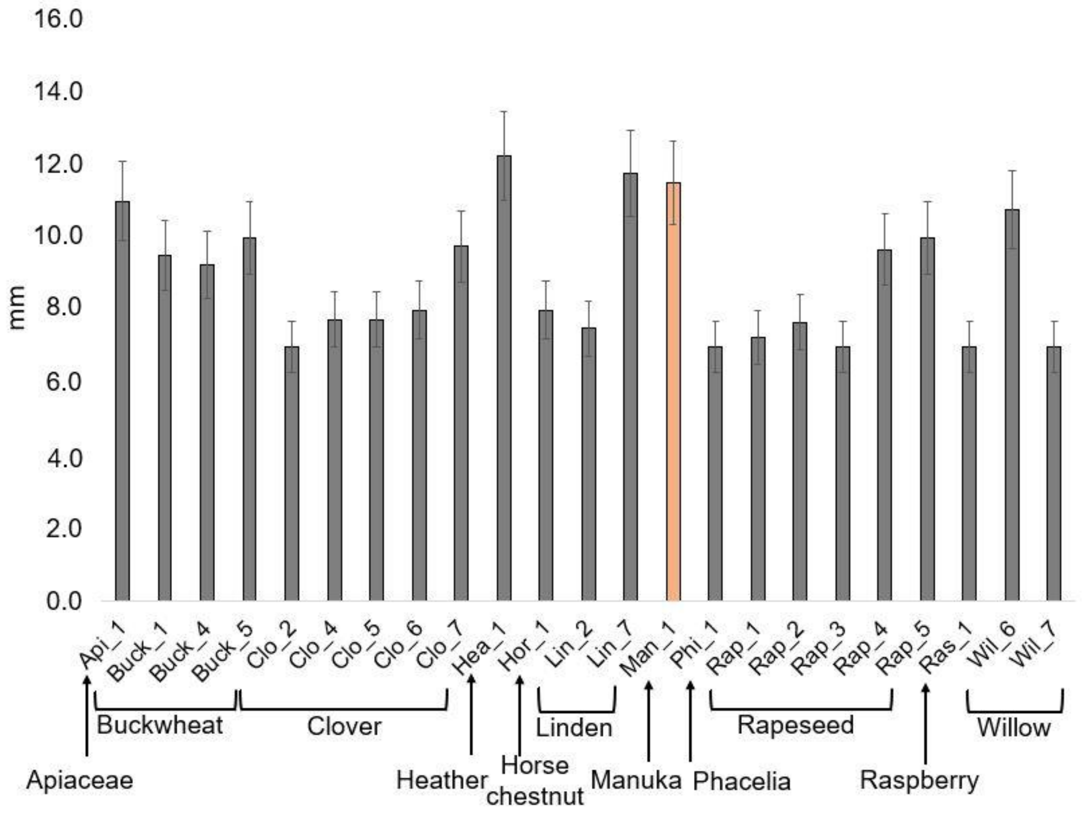


**(e)**

**Figure S1.** Inhibition zones of Latvian and Manuka honey samples for the antimicrobial activity with well-diffusion method. (**a**) *Staphylococcus aureus* (ATCC 25923); (**b**) *Methicillin-resistant staphylococcus aureus*; (**c**) *Pseudomona aeruginosa* (ATCC 14209); (**d**) *Escherichia coli* (ATCC 25922); (**e**) *Extended Spectrum Beta-Lactamase.*
