# Supplementary material for: Antimicrobial and Antibiofilm Properties of Latvian Honey against Causative Agents of Wound Infections
Source: Antibiotics (Basel). 2023 Apr 26;12(5):816. doi: 10.3390/antibiotics12050816 (PMC10215129; doi:10.3390/antibiotics12050816)
Supplement: Supplementary file 1 [file antibiotics-12-00816-s001.zip › Supplementary material Figure S2.pdf]

	Control	Apiaceae, Api1
**EC**	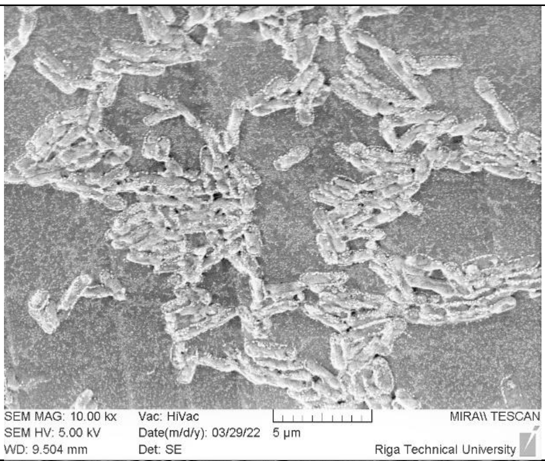	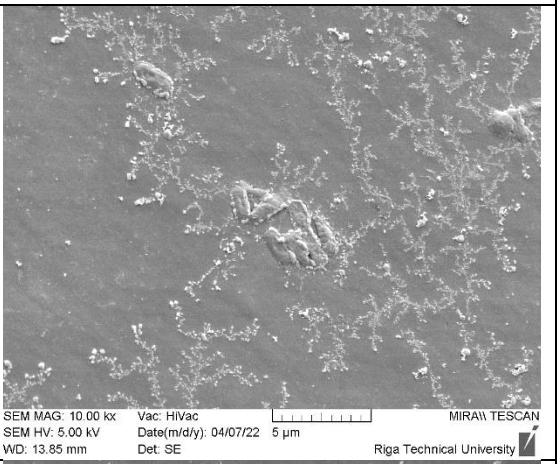
**ES**	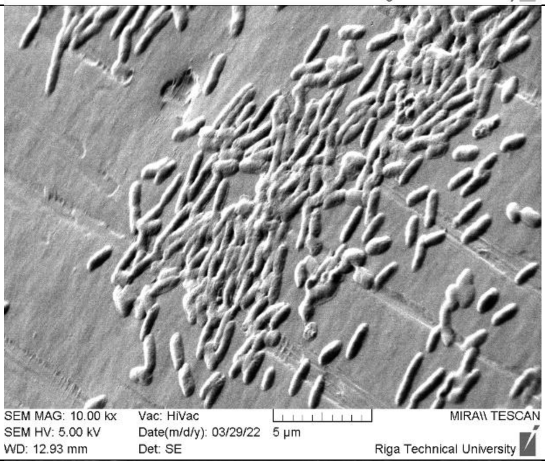	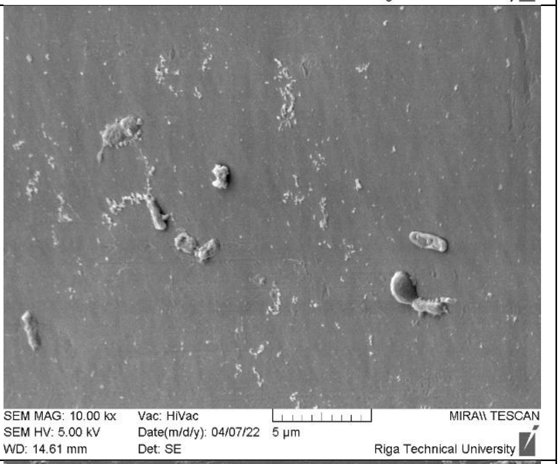
**PA**	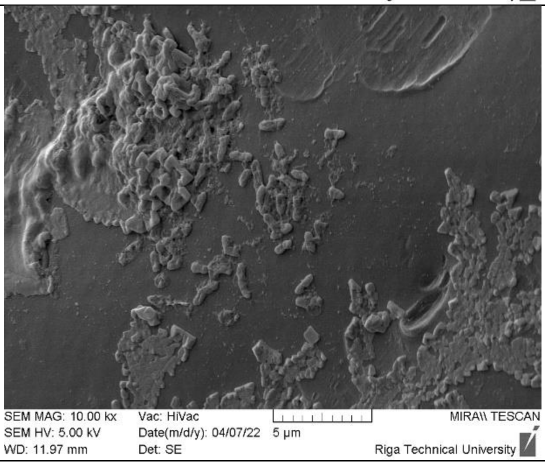	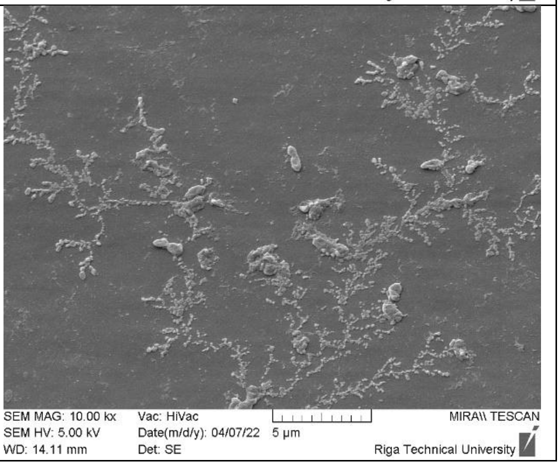

<table>
<tr><th></th><th>Control</th><th>Apiaceae, Api1</th></tr>
<tr><td>SA</td><td>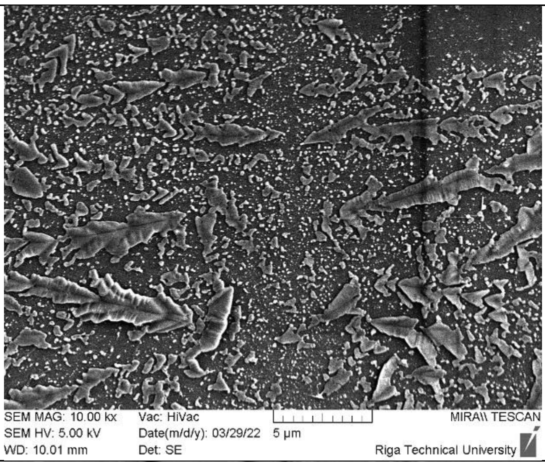
</td><td>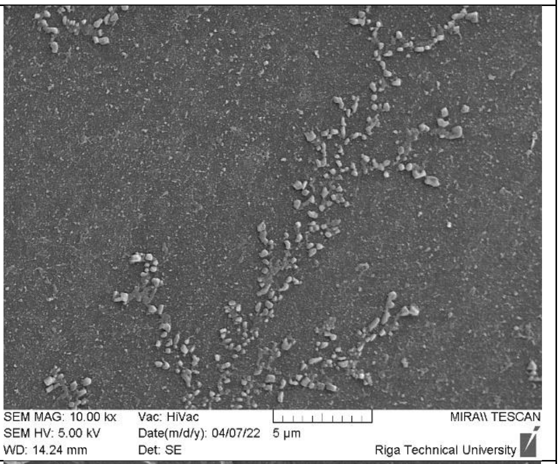
</td></tr>
<tr><td>MR</td><td>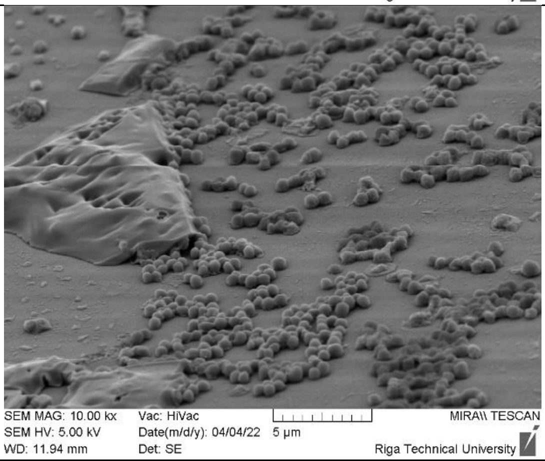
</td><td>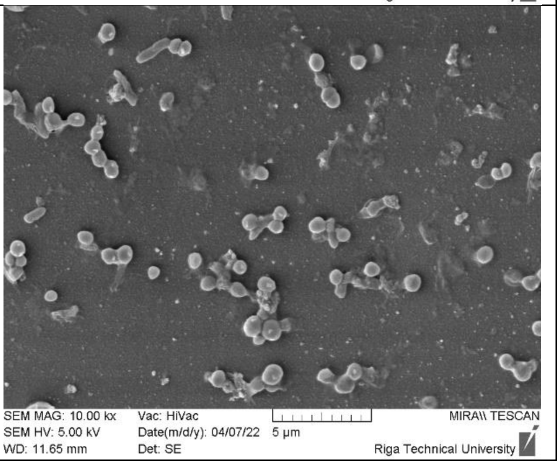
</td></tr>
</table>

	Control	Manuka
**EC**	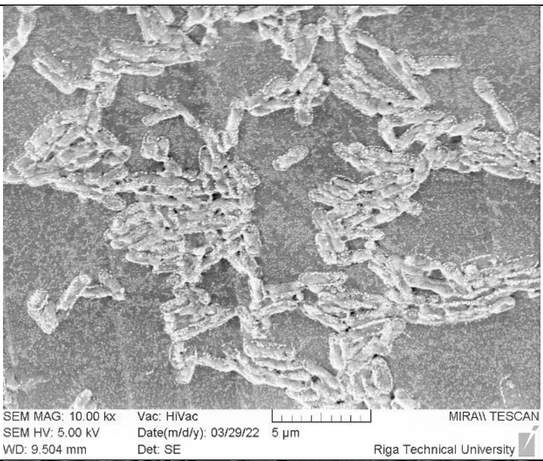	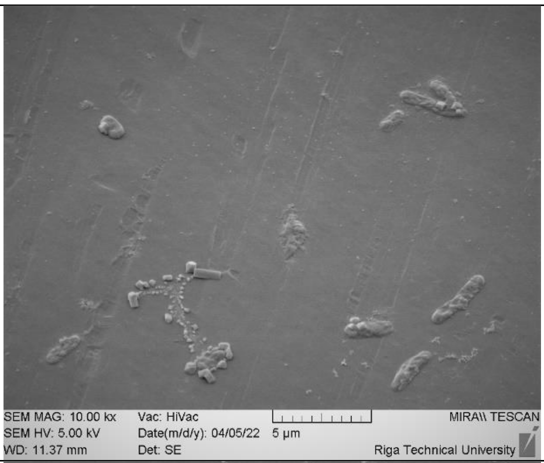
**ES**	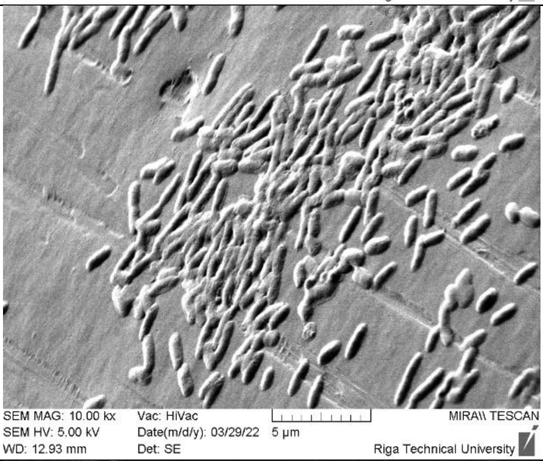	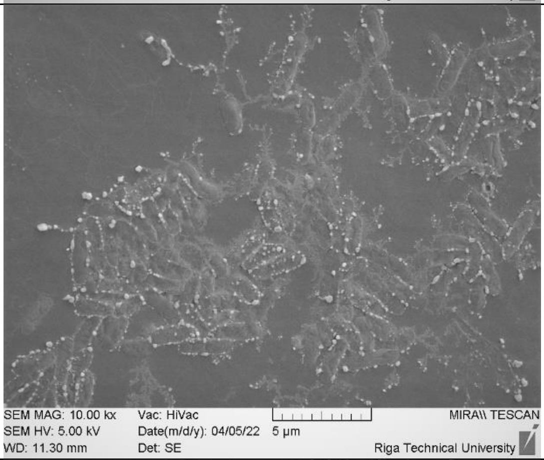
**PA**	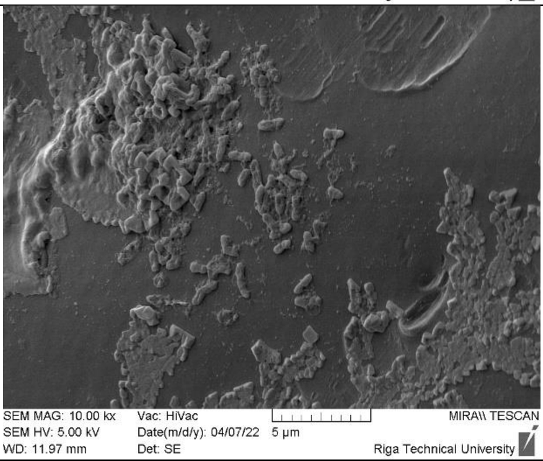	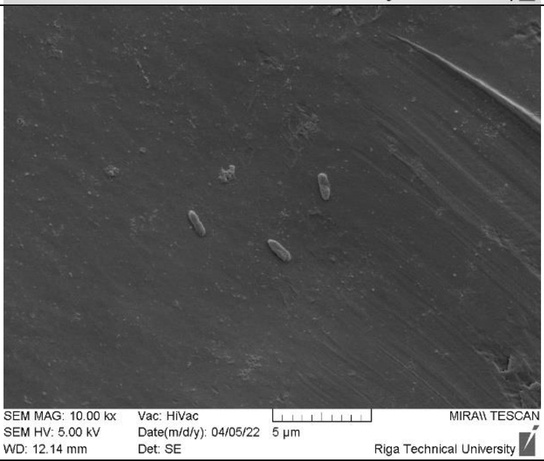

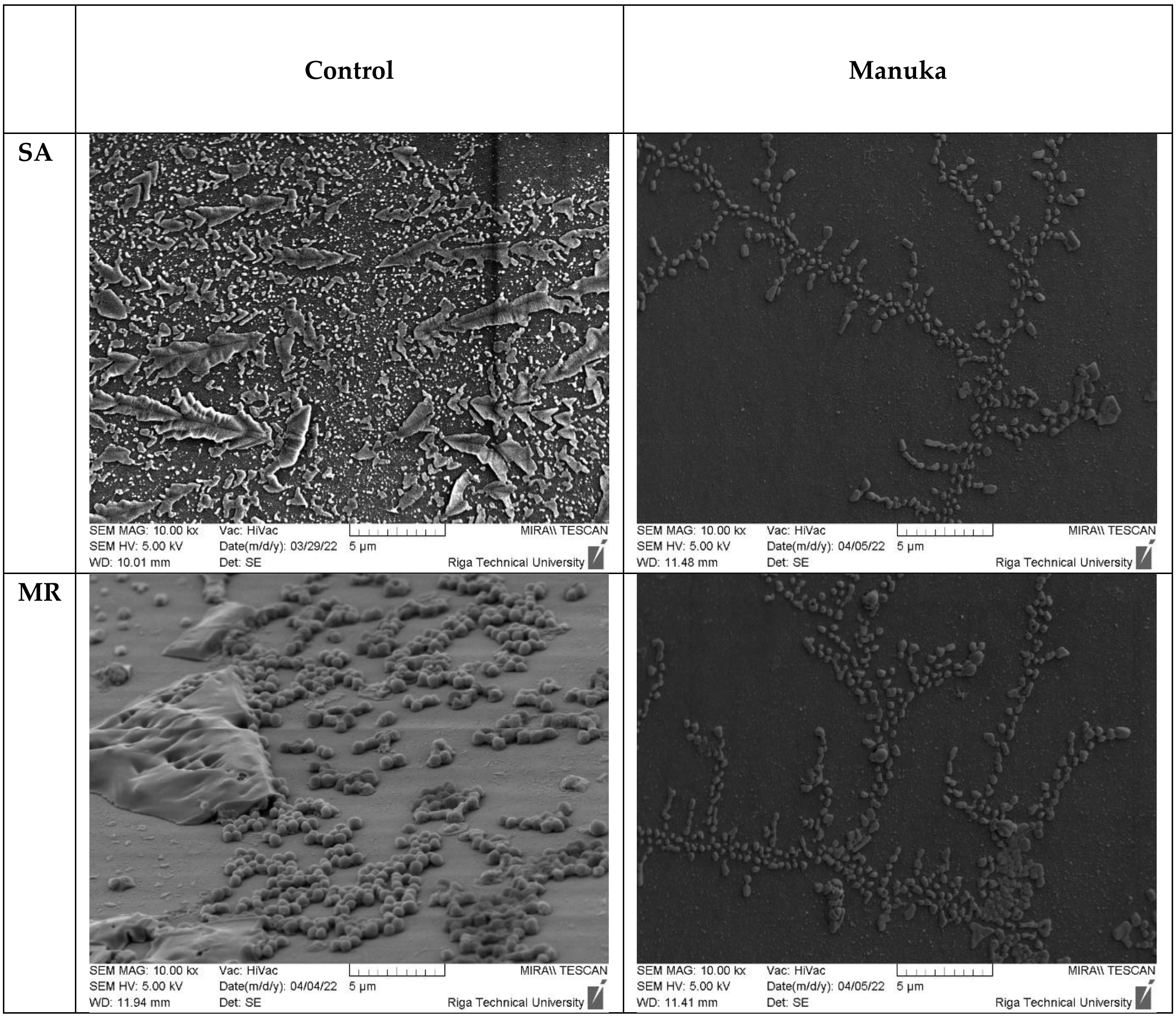

Figure S2. Scanning electron microscopic images of antibiofilm activity against Escherichia coli (EC) ATCC 25922, Extended-Spectrum Beta-Lactamases (ES), Pseudomonas aeruginosa ATCC 27853 (PA), Staphylo-coccus aureus (ATCC 29213) (SA) and Methicillin-resistant staphylococcus aureus (MR): Control samples of bacterial strains, Effect of Apiaceae (Api_1) honey, and effect of Manuka honey.
